# DSC3 expression is regulated by p53, and methylation of DSC3 DNA is a prognostic marker in human colorectal cancer

**DOI:** 10.1038/bjc.2011.28

**Published:** 2011-03-01

**Authors:** T Cui, Y Chen, L Yang, T Knösel, K Zöller, O Huber, I Petersen

**Affiliations:** 1 Institute of Pathology, University Hospital Jena, Ziegelmühlenweg 1, Jena 07743, Germany; 2 Institute of Biochemistry II, University Hospital Jena, Nonnenplan 4, Jena 07743, Germany

**Keywords:** DSC3, DNA methylation, p53, prognosis, colorectal cancer

## Abstract

**Background:**

Desmocollin 3 (DSC3), a member of the cadherin superfamily and integral component of desmosomes, is involved in carcinogenesis. However, the role of DSC3 in colorectal cancer (CRC) has not yet been established.

**Methods:**

Desmocollin 3 expression in CRC cell lines was analysed by RT–PCR and western blotting. Methylation status of DSC3 was examined by demethylation tests, methylation-specific PCR, and bisulphite sequencing (BS). The regulatory role of p53 was investigated by transfection.

**Results:**

Desmocollin 3 was downregulated in CRC cells at mRNA and protein levels. Desmocollin 3 expression was restored in five out of seven cell lines after 5-aza-2′-deoxycytidine (DAC) treatment. A heterogeneous methylation pattern was detected by BS in promoter region and exon 1 of DSC3. Methylation of DSC3 genomic sequences was found in 41% (41 out of 99) of primary CRC, being associated with poor prognosis (*P*=0.002). Transfection of p53 alone or in combination of DAC increased the DSC3 expression. Similarly, treatment with p53 inducer adriamycin alone or in combination with DAC enhanced DSC3 expression.

**Conclusions:**

DNA methylation contributes to downregulation of DSC3 in CRC cell lines. Methylation status of DSC3 DNA is a prognostic marker for CRC. P53 appears to have an important role in regulating DSC3 expression.

Colorectal cancer (CRC) is the third most frequently diagnosed cancer in men and women in the United States. With 655 000 deaths worldwide per year, this cancer type is also the third most common leading cause of cancer-related death in the western world (Cunningham *et al*, 2010). Improved treatment and efficient surveillance contributed to control the disease; however, the overall 5-year survival rate of CRC is still poor (Hamilton and Aaltonen, 2000).

Colorectal cancer arises through multiple genetic changes, including mutations of APC, TP53, RAS, and RAF (Kouzminova *et al*, 2010). However, epigenetic alternations such as DNA methylation also have an important role through the silencing of cancer-related genes in CRC (Jones and Baylin, 2007). Cancer cells often have both a loss of global methylation and a gain of methylation at the promoter of selected CpG islands, resulting in the silencing of hundreds of genes (Jones and Baylin, 2002; Issa, 2004). Indeed, DNA methylation has been proven to be a useful marker of disease risk, progression, and prognosis in various malignancies including CRC. Recently, a specific DNA methylation inhibitor 5-aza-2′-deoxycytidine (DAC, also called decitabine) was approved by the FDA for the treatment of the haematologic malignancy myelodysplastic syndrome in the United States of America (Lemaire *et al*, 2008).

P53 is a tumour suppressor protein that acts as a checkpoint in the cell cycle, either preventing or initiating programmed cell death (Buckbinder *et al*, 1995). Loss of normal p53 function occurs in many human tumours and primarily is caused by mutation or deletion of the p53 gene (Amaral *et al*, 2010). Many p53 target genes have been reported to be important for maintenance of normal cellular behaviour, and a loss of p53 regulation may cause abnormal expression of these genes. Interestingly, several targets of p53 are silenced by DNA methylation (Wales *et al*, 1995; Suzuki *et al*, 2000; Christoph *et al*, 2006).

Desmosomes represent lateral punctate cell–cell junctions linked with the intermediate filament apparatus of vertebrate cells. Members of the desmosomal cadherin family, including democollins 1–3 (DSCs) and desmogleins 1–4 (DSGs), are found primarily in epithelial cells where their extracellular domains constitute the adhesive interface, and together with cytosolic plaque proteins are required for assembly and maintenance of desmosome (Garrod *et al*, 2002). Impaired desmosomal protein function is associated with multiple diseases (Huber, 2003). One of the interesting functions of desmosomal proteins related to cancer is their ability to inhibit cell motility (Tselepis *et al*, 1998). Indeed, evidence supporting the involvement of desmosomal proteins in carcinogenesis is growing. For example, DSG2 was found to be overexpressed in skin cancer (Kurzen *et al*, 2003), whereas DSG 3 was upregulated in head and neck cancer and lung cancer (Chung *et al*, 2004; Chen *et al*, 2007; Fukuoka *et al*, 2007). By contrast, DSC3 was downregulated in breast cancer due to promoter hypermethylation (Oshiro *et al*, 2005).

In this study, we analysed the DSC3 expression, investigated the methylation status of DSC3 genomic sequences, and explored the role of p53 in the regulation of DSC3 expression in CRC.

## Materials and methods

### Cell lines and cell culture

Human normal colon cells (CCD-33Co) were purchased from the American Type Culture Collection (ATCC, Rockville, MD, USA). Human colon cancer cell lines (HT-29, LoVo, Caco-2, WiDr, SW480, HCT116, and HRT-18) were purchased from ATCC. CX-2 was obtained as a gift from Dr Antje Siegert (Institute of Pathology, Charité Berlin). HT-29, SW480, and HCT116 were grown in Leibovitz 15 medium supplemented with 10% FCS and 1% glutamine. Caco-2, WiDr, CX-2, and HRT-18 were cultured in RPMI1640 medium supplemented with 10% (v/v) FCS and 1% (w/v) glutamine. LoVo was cultured in DMEM medium including 10% (v/v) FCS, 1% (w/v) glutamine, and 4.5% (w/v) glucose.

### RNA extraction and real-time RT–PCR

Total RNA was extracted from cells using the Trizol reagent (Invitrogen, Darmstadt, Germany) according to manufacturer's instructions. One microgram of total RNA was incubated with RQ1 RNase-free DNase (Promega, Madison, WI, USA) for 30 min at 37°C to eliminate genomic DNA contamination. Messenger RNA was reverse-transcribed by 15 U ThermoScript RT (Invitrogen) from DNase-treated RNA in the presence of 1 × RT buffer, 100 mM DTT, 20 U RNase inhibitor, and 10 mM dNTPs, using the random hexamer primers supplied in the kit.

Expression of DSC3 was analysed by real-time PCR. Real-time PCR was performed in 0.1 ml tubes on the Rotor-Gene 6000 (Qiagen, Hilden, Germany) in the presence of the FastStart Universal SYBR Green Master (Roche, Mannheim, Germany). Twenty-five nanograms of RNA were used for PCR amplification with the following primer pairs for DSC3: 5′-GAAAGTAGTAGACCTGGTACT-3′ (forward), 5′-ACGCCTGTGCTGGGATGCA-3′ (reverse) and for p53: 5′-GGTGGTGCCCTATGAGCCG-3′ (forward), 5′-TCCTCTGTGCGCCGGTCTC-3′ (reverse). As an internal control, glyceraldehyde-3-phosphate dehydrogenase (GAPDH) was amplified using the primer pair: 5′-TCAAGGGCATCCTGGGCTACA-3′ (forward) and 5′-CCAGCCCCAGCGTCAAAGGT-3′ (reverse). The relative expression value of DSC3 to GAPDH in each sample was calculated and compared.

### Western blot analysis

Protein from cell lysates was isolated, and 20 *μ*g of protein were used for western blot analysis according to standard protocols. Monoclonal anti-desmocollin-3 antibody (Acris, Herford, Germany) was used at a 1 : 100 dilution. Signal was visualised with peroxidase-conjugated polyclonal rabbit anti-mouse antibody (1 : 1000, DAKO, Hamburg, Germany) and ECL Plus Western Blotting Detection Reagents (GE Healthcare, München, Germany).

### Bisulphite modification and methylation-specific PCR

Bisulphite conversion of genomic DNA was carried out using an EZ DNA Methylation kit (ZYMO Research, Freiburg, Germany). This process converts unmethylated cytosine to uracil, whereas methylated cytosine remains unchanged. Methylation-specific PCR (MSP) was then carried out to determine the methylation status of DSC3 in colon cancer cell lines and primary colon tumour tissues. Bisulphite-modified DNA was used as a template for PCR with primers specific for methylated or unmethylated alleles.

The primer sequences were as follows: 5′-GGGTGTGTTAGAGATTGTTTTTTT-3′ (forward) and 5′-AAACAACTTCACTTCTAAAACCAAA-3′ (reverse) for amplification of unmethylated allele, 5′-GTGCGTTAGAGATCGTTTTTTC-3′ (forward) and 5′-AAACAACTTCACTTCTAAAACCGAA-3′ (reverse) for amplification of methylated allele. PCR conditions were as follows: 94°C 1 min, 60°C 45 s, and 72°C 45 s, for 40 cycles, with initial denaturation at 95°C for 15 min and final elongation at 72°C for 7 min. The PCR products were visualised on a 1.5% (w/v) agarose gel stained with ethidium bromide.

### Bisulphite sequencing

Bisulphite-treated DNA was subjected to PCR amplification. To determine the methylation pattern of the CpG islands within the DSC3 promoter and exon 1, two pairs of primers for bisulphite sequencing (BS) were designed. These primers were specific to the modified template but did not contain any CpG site in their sequences, so they could amplify both methylated and unmethylated sequences. The primer pair BSP-PF (5′-TTGGGGTTTTGTATTGAGATGTA-3′) and BSP-PR (5′-AACCTACCCCTCTACTCCCC-3′) were used to amplify the CpG island in the promoter region with an expected product of 210-bp. The second primer pair BSP-EF (5′-GAGGGTTAGTAGTAGATGTAGGTA-3′) and BSP-ER (5′-AACAAAACCTAAAAAAAACCAA-3′) were designed to amplify the CpG island in the region of exon 1 and the size of the product was 189-bp. Hot Start PCR (PEQLAB, Erlangen, Germany) was carried out to amplify the CpG island of DSC3 with the following conditions: 95°C 15 min, 40 cycles of 95°C 1 min, 60°C 45 s, and 72°C 45 s, with a final extension at 72°C for 7 min. PCR products were purified by using a PCR purification kit (ZYMO Research), and 150 ng of purified PCR products were applied for direct sequencing by capillary electrophoresis (Agowa, Berlin, Germany).

### Drug treatment and transient transfection

For demethylation tests, the colon cancer cell lines HT-29, LoVo, WiDr, SW480, CX-2, HCT116, and HRT-18 were plated and cultured in 10 cm dishes. At 50% confluence, 10 *μ*M 5-aza-2′-deoxycytidine (DAC; Sigma Chemical Co., St Louis, MO, USA) was added to the medium on days 0, 2, and 4. Cells were harvested for total RNA isolation and RT–PCR analysis. To determine the effect of p53 on DAC-induced expression of DSC3, CX-2, WiDr, and HRT-18, cells (1 × 10^5^ cells per well in 12-well plates) were treated with low dose of DAC (5 *μ*M) on days 0 and 2 or without DAC. On day 3, cells were transfected with wild-type p53 expression vector (gift from Dr Ying Liu, Oxford University, UK) by using lipofectamin 2000 (Invitrogen) and incubated for another 48 h. To investigate the regulatory role of endogenous p53 on DSC3, CX-2, WiDr, and HRT-18, cells (1 × 10^5^ cells per well in 12-well plates) were first treated with ADR at different concentrations from 0.1 to 1.0 *μ*g ml^−1^ for 24 h before analysis of p53 expression. Then, the three cell lines were treated with 1 *μ*M DAC on days 0 and 1, and subsequently on day 2, ADR was added to a final concentration of 0.5 *μ*g ml^−1^ and incubated for 24 h before harvesting of cells for analysis of DSC3 expression.

### Clinical samples and genomic DNA isolation

In total, 99 tumour specimens (paraffin tissues) from patients with CRC were included for this study (Table 1). All of these patients were undergoing surgical operation at the Department of Surgery of the University Hospital Jena. No adjuvant radiotherapy or chemotherapy was administered before surgery. The study was approved by the local ethical committee.

All haematoxylin and eosin (H and E) sections were reviewed by a pathologist (T Knösel). Suitable areas for genomic DNA isolation were marked on H and E sections. Genomic DNA was extracted by using a QIAamp DNA Mini Kit (Qiagen). In order to minimise the contamination of normal mucosa, manual microdissection was performed before DNA isolation.

### Immunohistochemistry

Immunohistochemistry analyses were performed with tumour specimen obtained from the 99 patients with primary CRC, for which methylation status has been investigated. Tissue sections were dewaxed with xylene and gradually hydrated. Antigen retrieval was performed by treatment in a pressure cooker for 6 min. The monoclonal anti-desmocollin-3 antibody (Acris) used at a 1 : 50 dilution was incubated at room temperature for 1 h. Detection took place according to the manufacturer's instructions (LSAB 2-kits, DAKO). All slides were read by two pathologists (T Knösel and I Petersen) who were blinded to the clinical information. Immunohistochemistry was scored semiquantitatively as negative (score 0), weak (score 1), moderate (score 2), or strong (score 3) as previously described (Chen *et al*, 2003). For statistical evaluation, score 0 was considered as negative, whereas scores 1, 2, and 3 together were positive.

### Statistical analysis

Kaplan–Meier survival curves were constructed for statistical significance with the log-rank tests. All *P*-values were calculated two-sided. Difference was considered statistically significant when *P-*value was 0.05 or less. The statistical analysis was performed by using the software package SPSS 13.0 (SPSS, Chicago, IL, USA).

## Results

### Expression analysis of DSC3 in colon cancer cell lines

**Figure 1:**
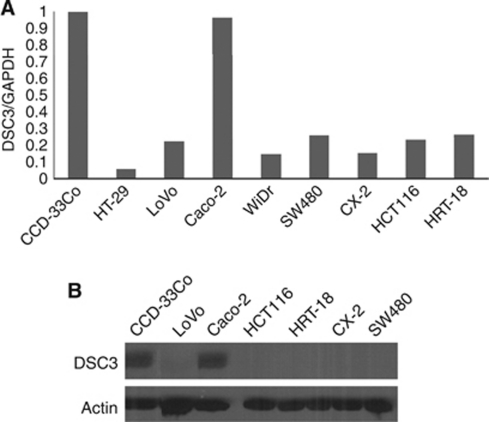
DSC3 expression analysis in CRC cell lines. (**A**) Desmocollin 3 mRNA expression was analysed by real-time RT–PCR showing that DSC3 was downregulated in seven out of eight cancer cell lines. (**B**) In consistence with mRNA expression, western blotting revealed decreased DSC3 protein expression in CRC cell lines. Actin was used as loading control.

We performed real-time RT–PCR to analyse the expression of DSC3 in eight colon cancer cell lines together with one normal colon cell line CCD-33Co. Compared with CCD-33Co, DSC3 mRNA expression was found downregulated in seven colon cancer cell lines, except Caco-2 (Figure 1A). Protein expression was analysed by western blotting showing decreased expression of DSC3 in cancer cell lines (Figure 1B).

### Restoration of DSC3 mRNA expression by 5-aza-2′-deoxycytidine

**Figure 2:**
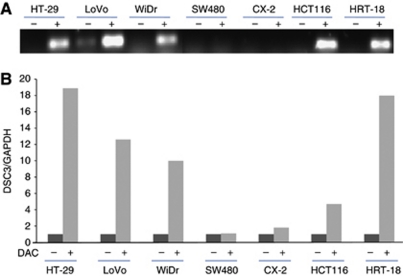
Demethylation tests in CRC cell lines. (**A**) Semiquantitative RT–PCR and (**B**) real-time RT–PCR showed that after treatment with 10 *μ*M DAC for 96 h, DSC3 mRNA expression was upregulated. (−)=untreated; (+)=treated with DAC.

To explore the epigenetic regulation of DSC3, seven colon cancer cell lines including HT-29, LoVo, WiDr, SW480, CX-2, HCT116, and HRT-18 were selected for the demethylation tests. After treatment with 10 *μ*M DAC for 96 h, DSC3 mRNA expression was restored in five (HT-29, LoVo, WiDr, HCT116, and HRT-18) out of seven cell lines. While in the other two cell lines (SW480 and CX-2), no restoration of DSC3 expression was detectable (Figures 2A and B).

### Analysis of DSC3 methylation status in colon cancer cell lines

**Figure 3:**
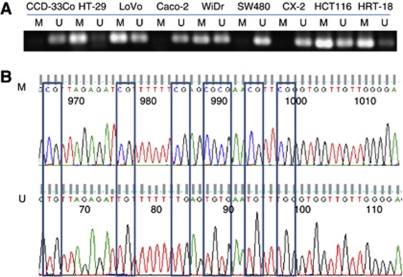
Methylation status of DSC3 DNA in CRC cell lines. (**A**) Methylation status of DSC3 DNA was detected by MSP in eight CRC cell lines. The DSC3 promoter region was unmethylated in the DSC3-positive cell line Caco-2 as well as in two DSC3-negative cell lines SW480 and CX-2, while in the other five DSC3-negative cell lines, the promoter of DSC3 was methylated. (**B**) Sequencing results of MSP. M=methylated product; U=unmethylated product.

The methylation status of DSC3 was determined by MSP in eight colon cancer cell lines. Methylation-specific PCR primers were designed in the region around the transcription start site of the DSC3 gene. Methylation-specific PCR showed that DSC3 DNA was methylated in cell line HT-29, LoVo, WiDr, HCT116, and HRT-18, but completely unmethylated in cell line SW480 and CX-2 (Figure 3A). This result was in good agreement with the demethylation tests. The reliability of the MSP results was verified by direct DNA sequencing (Figure 3B).

**Figure 4:**
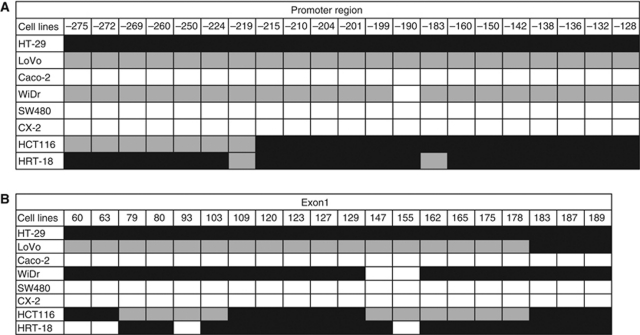
Methylation status of CpG sites in (**A**) promoter region and (**B**) exon 1 of DSC3. Black square: methylated CpG site; Grey square: partially methylated CpG site; White square: unmethylated CpG site.

To confirm the MSP results and further evaluate the methylation status of DSC3 in CRC cell lines, BS was performed for 21 CpG sites (−275, −272, −269, -260, −250, −224, −219, −215, −210, −204, −201, −199, −190, −183, −160, −150, −142, −138, −136, −132, and −128) of the promoter region. Consistent with results of our MSP analysis, a high level of methylation was found in five out of seven cell lines with downregulated DSC3 expression (HT-29, LoVo, WiDr, HCT116, and HRT-18), except SW480 and CX-2 (Figure 4A). In exon 1, we evaluated the methylation status of DSC3 DNA in 20 CpG sites (+60, +63, +79, +81, +93, +103, +109, +120, +123, +127, +129, +147, +155, +162, +165, +175, +178, +183, +187, and +189). Again, in these five cell lines, DSC3 was highly methylated (Figure 4B). As expected, in the cell line Caco-2 with endogenous expression of DSC3, no methylation of DSC3 was detected.

### Methylation of DSC3 predicts poor clinical outcome

**Figure 5:**
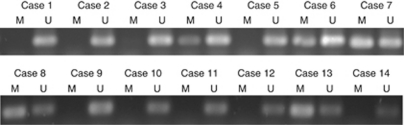
Examples of MSP of DSC3 DNA from patients with primary CRC. M=methylated product; U=unmethylated product.

**Figure 6:**
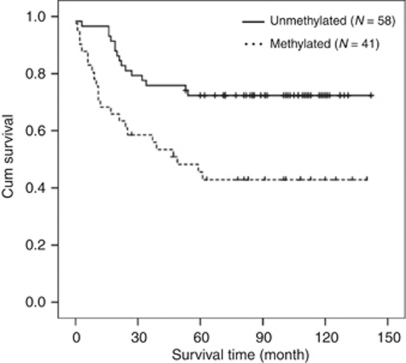
Methylation of DSC3 DNA predicted clinical outcome in primary colorectal cancer. Kaplan–Meier curves showed that patients whose tumours with methylated DSC3 DNA had shorter survival in comparison with patients whose tumours with unmethylated DSC3 DNA.

The specificity of MSP in CRC cell lines encouraged us to analyse the methylation status of DSC3 DNA in 99 primary colorectal tumours by using the same primer pairs. Methylation of DSC3 DNA was detected in 41 out of 99 tumours (41.4%). Examples of MSP analysis in primary tumours are shown in Figure 5. Methylation of DSC3 DNA was found in 23 out of 39 (59%) patients who had a survival time <5 years, whereas in patients with survival time >5 years, only 30% of the patients (18 out of 60) harboured DSC3 DNA methylation, reaching statistical significance (*P*=0.004; Table 2). When we further analysed the effect of methylation on clinical outcome by Kaplan–Meier analysis, we found that tumours with methylated DSC3 DNA were significantly correlated to a worse clinical outcome than unmethylated tumours (*P*=0.002, Figure 6). However, the methylation status was not linked to any of clinical–pathological parameters including age, gender, size of tumour, tumour grading, and tumour stage in these patients.

We also analysed the DSC3 protein expression in these 99 primary tumours by immunohistochemistry. It turned out that DSC3 protein expression was neither associated with clinical–pathological parameters nor patient survival (data not shown).

### Effect of p53 on regulating DSC3 mRNA expression

**Figure 7:**
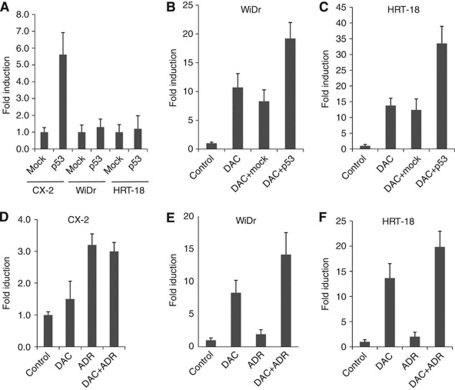
Transcriptional activation of DSC3 by p53. (**A**) Induction of DSC3 mRNA expression after transfection of the three colon cancer cell lines CX-2, WiDr, and HRT-18 with an expression vector encoding wild-type p53 was measured by quantitative RT–PCR. Mock transfectants (empty vector) were used as control. (**B** and **C**) P53-induced DSC3 expression was enhanced after DAC treatment in the cell lines WiDr and HRT-18. Parental cells were used as control. (**D**) Induction of DSC3 transcription by ADR, but not DAC, in the cell line CX-2. (**E** and **F**) Synergistic effect of combined ADR+DAC treatment on WiDr and HRT-18 cells, respectively.

In breast cancer, p53 was reported to be an upstream factor regulating expression of DSC3 (Oshiro *et al*, 2003). To investigate a potential mechanistic link between p53 and DSC3 expression in colon cancer cells, we selected three cell lines including CX-2 (DSC3-unmethylated, p53-mutant), WiDr (DSC3-methylated, p53-mutant), and HRT-18 (DSC3-methylated, p53-wild type) for transfection with a wild-type p53 expression vector. A successful transfection was confirmed by real-time RT–PCR analysis and western blotting demonstrating the upregulation of p53 in the transfected cell lines, compared with parental cells and mock transfectants (Supplementary Figure S1). Exogenous expression of p53 alone induced expression of DSC3 in the unmethylated cell line CX-2, but not in the methylated cell lines WiDr and HRT-18 (Figure 7A). In contrast, transfection of p53 in combination with DAC treatment resulted in upregulation of DSC3 expression in the two methylated cell lines (Figures 7B and C). We further analysed the effect of endogenous p53 on DSC3 expression by treatment of CX-2, WiDr, and HRT-18 with adriamycin (ADR), an inducer of endogenous p53 expression. Induction of p53 mRNA expression was achieved in the three cell lines by ADR modification with different concentrations (Supplementary Figure S2A), while increased expression of p53 was only observed in the cell line HRT-18 (Figure S2B) but not in the p53-mutant cell lines CX-2 and WiDr (Supplementary Figure S2B). The DSC3 expression was analysed by real-time RT–PCR. It turned out that in the unmethylated cell line CX-2, ADR alone could markedly enhance the expression of DSC3 (Figure 7D). In contrast, in the methylated cell lines WiDr and HRT-18, ADR alone could only slightly upregulate the DSC3 expression. When ADR was combined with DAC, a synergistic effect on upregulation of DSC3 expression was observed in WiDr and HRT-18 (Figures 7E and 7F).

## Discussion

Desmocollin 3 is a transmembrane glycoprotein and a member of the cadherin superfamily of calcium-dependent cell–cell adhesion molecules. Desmocollin 3 was found to be downregulated in breast and oral cancer, and gene silencing in breast cancer was caused by promoter hypermethylation (Oshiro *et al*, 2005). However, so far little is known about the role of DSC3 in CRC.

In our study, first, we analysed the mRNA expression of DSC3 in eight colon cancer cell lines and found that the expression of DSC3 was significantly downregulated in seven out of eight cell lines. Western blot analysis confirmed these results on the protein level.

Next, we explored the mechanism responsible for the downregulation of DSC3 expression in CRC. Gene expression is modulated by genetic and/or epigenetic mechanisms. So far, no genetic changes including mutation, deletion, and gene rearrangement of DSC3 have been found in cancer, and except for the detailed description of methylation patterns in breast cancer, no methylation analysis of DSC3 in any other tumour entity has been reported. In our study, demethylation of DSC3 by using the pharmaceutical agent DAC restored the DSC3 expression in seven CRC cell lines lacking endogenous DSC3 expression. The DSC3 DNA methylation status in the promoter region and exon 1 of the gene was analysed by BS. In five out of seven cancer cell lines, DSC3 DNA methylation was detectable in 21 CpG sites of the promoter region and in 20 CpG sites of exon 1. The methylation pattern was however heterogeneous with full methylation of DSC3 in cell line HT-29, and incomplete or partial methylation in LoVo, WiDr, HCT116, and HRT-18. Treatment of SW480 and CX-2 with DAC failed to restore the expression of DSC3, and BS showed that DSC3 DNA was not methylated, suggesting that other mechanisms might be involved in downregulation of DSC3 in these two cell lines. These results suggest that DNA hypermethylation contributes to the gene silencing of DSC3 in a multitude of colon cancer cells.

The methylation pattern of DSC3 in cell lines raised the question about the methylation status of DSC3 in primary CRC. Therefore, we performed MSP with genomic DNA from 99 tissues of primary CRC. It turned out that patients with a survival time <5 years showed higher rate of methylation (56.1%). On the contrary, patients who had a survival time of >5 years harboured a lower rate of methylation (30%). Similarly, tumours with methylated DSC3 DNA were significantly correlated to a worse clinical outcome in comparison with unmethylated tumours. These observations indicated that methylation of the promoter region was significantly associated with poor clinical outcome, and methylation status of DSC3 may represent a potential prognostic marker for patients with CRC.

The regulatory mechanisms controlling DSC3 expression are not fully understood. Desmocollin 3 is a p53 response gene and addition of wild-type p53 was found to be sufficient to induce expression of DSC3 in breast cancer (Klus *et al*, 2001). Thus, it was of great interest to investigate whether this pathway is also active in CRC cells. We found that exogenous expression of p53 increased the DSC3 expression in the cell line harbouring no DSC3 DNA methylation. In cancer cell lines with methylated DSC3 DNA, induction of DSC3 expression after transfection of p53 was only achieved in the presence of the demethylating agent DAC. We further tested the role of p53 by inducing its endogenous expression using ADR. Adriamycin (also called doxorubicin) is an anticancer drug used in treatment of breast, ovarian, bladder, and gastric cancers. It has been reported that ADR activates p53 expression and induces p53 target genes during damaged DNA repair (Adachi *et al*, 2004; Maruyama *et al*, 2006; Suzuki *et al*, 2010). We found that induction of endogenous p53 by ADR markedly enhanced the expression of DSC3 in the cell line CX-2 with unmethylated DSC3 DNA, while only a slight increase in the expression of DSC3 was detectable in WiDr and HRT-18 cells revealing methylated DSC3 DNA. However, in these two cell lines, combined treatment with ADR and DAC induced DSC3 expression. These results suggest that combining a DNA methyltransferase inhibitor with drugs that induce p53-dependent growth inhibition may represent a useful therapeutic approach for the treatment of CRC. In fact, DAC has been considered as a part of combination therapy with other anticancer agents to treat ovarian, breast, prostate, gastric, lung, pancreatic, and colon cancers through the relief of DNA hypermethylation (Shames *et al*, 2007).

In summary, DSC3 is downregulated in CRC cell lines by DNA methylation. This is the first study reporting a detailed methylation status of DSC3 DNA in CRC cells. Analysis of the methylation status of DSC3 DNA may be useful to predict clinical outcomes in patients with primary CRC. Future studies are necessary to determine the functional role of DSC3 in CRC initiation and progression.

## Figures and Tables

**Table 1:** Study cohort

	**DSC3 (M)**	**DSC3 (U)**	***P***
*Age*			
⩽63	16 (16.2%)	32 (32.3%)	0.113
>63	25 (25.3%)	26 (26.3%)	
			
*Gender*			
Male	22 (22.2%)	28 (28.3%)	0.769
Female	23 (23.2%)	26 (26.3%)	
			
*Tumour differentiation*			
G1/2	18 (19.6%)	35 (38%)	0.154
G3	19 (20.7%)	20 (21.7%)	
			
*Stage*			
pT1/2	3 (3%)	12 (12.1%)	0.090
pT3/4	38 (38.4%)	46 (46.5%)	
			
*Nodal status*			
pN0	17 (17.2%)	31 (31.3%)	0.240
pN1/2	24 (24.2%)	27 (27.3%)	

Abbreviations: DSC3=desmocollin-3; M=methylated; N=unmethylated.

**Table 2:** Correlation between DSC3 methylation and survival time (*P*-value^*^)

	**⩽5 years**	**>5 years**	***P***
DSC3 Methylated	23 (23.2%)	18 (18.2%)	0.004
DSC3 Unmethylated	16 (16.2%)	42 (42.4%)	

Abbreviation: DSC3=desmocollin-3. ^*^P-values are statistically significant.
