# Supplementary material for: DSC3 expression is regulated by p53, and methylation of DSC3 DNA is a prognostic marker in human colorectal cancer
Source: Br J Cancer. 2011 Mar 1;104(6):1013–9. doi: 10.1038/bjc.2011.28 (PMC3065270; doi:10.1038/bjc.2011.28)
Supplement: Supplementary Figures [file bjc201128x1.ppt]

## Slide 1
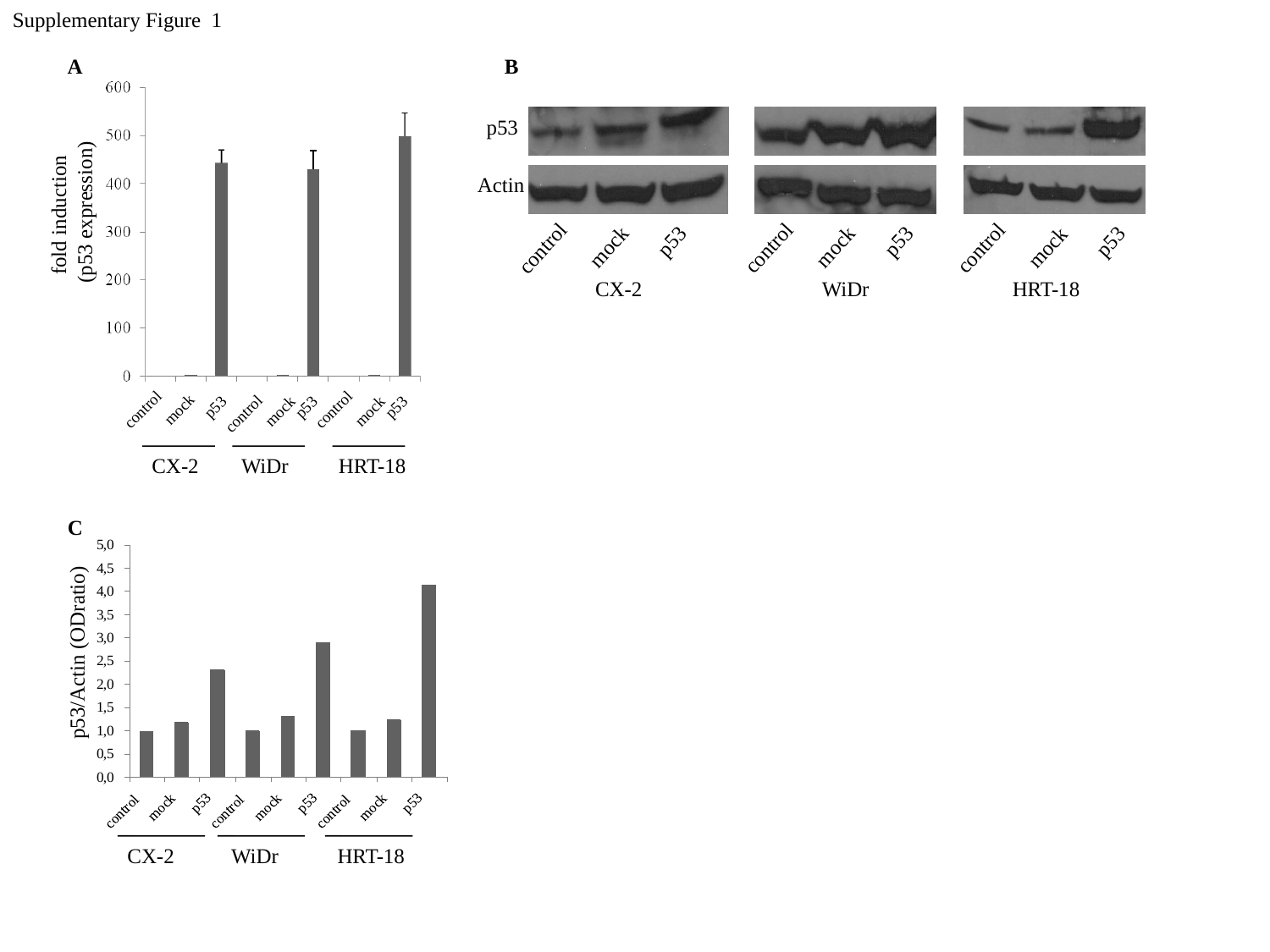

Supplementary Figure 1
A
fold induction
(p53 expression)
p53
p53
p53
p53
mock
mock
mock
control
control
control
CX-2
WiDr
HRT-18
B
p53
p53
p53
p53
control
control
control
mock
mock
mock
CX-2
WiDr
HRT-18
Actin
C
p53/Actin (ODratio)
CX-2
WiDr
HRT-18

## Slide 2
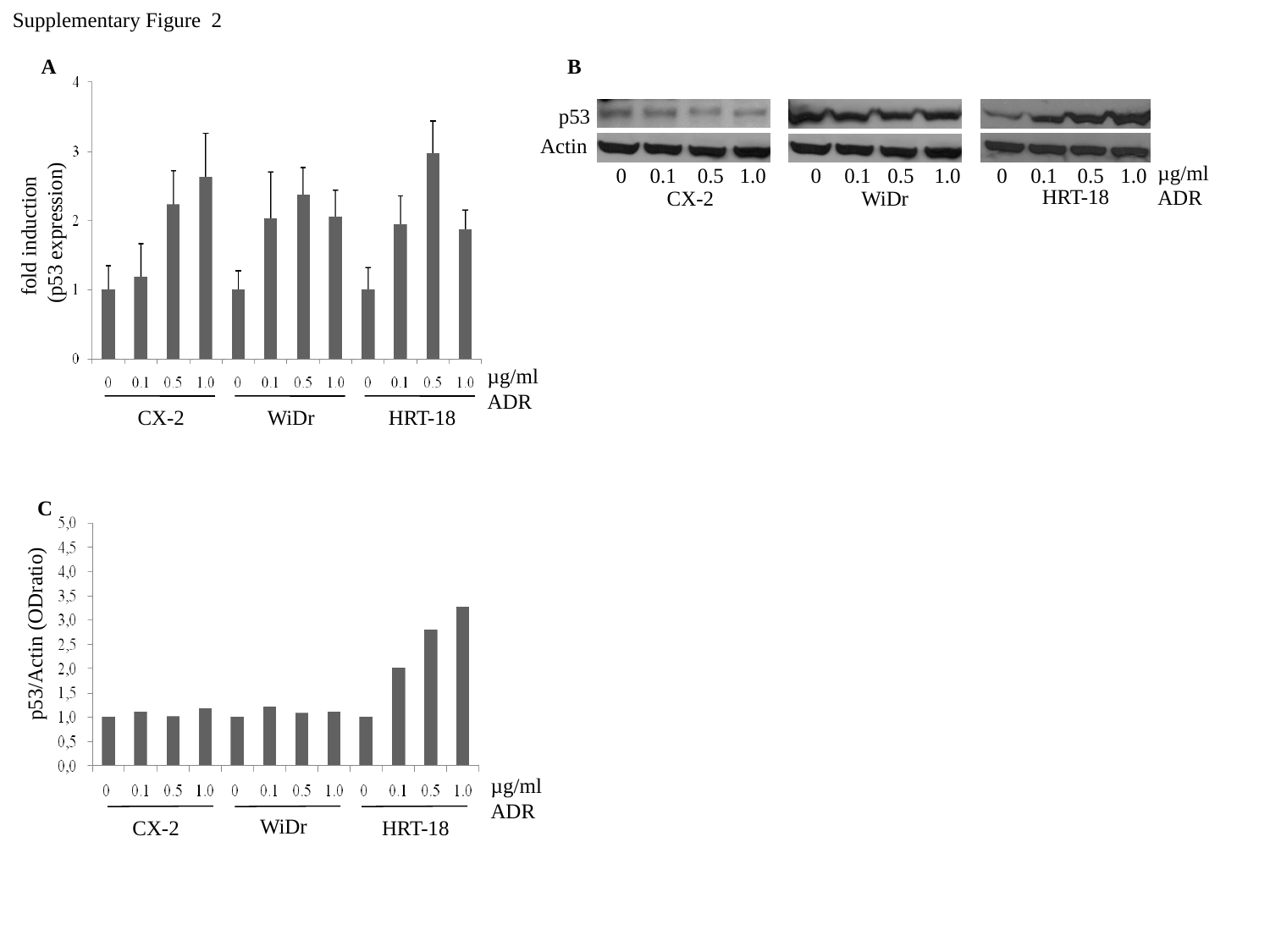

Supplementary Figure 2
A
CX-2
WiDr
HRT-18
B
p53
Actin
µg/ml ADR
0
0.1
0.5
1.0
0
0.1
0.5
1.0
0
0.1
0.5
1.0
HRT-18
CX-2
WiDr
fold induction
(p53 expression)
µg/ml ADR
C
p53/Actin (ODratio)
µg/ml ADR
WiDr
CX-2
HRT-18
